# The Correlation between Sex Hormone-Binding Globulin and Clinical Characteristics According to Anti-Müllerian Hormone in Women with Regular Menstrual Cycles: A Prospective Study

**DOI:** 10.3390/jpm14030274

**Published:** 2024-02-29

**Authors:** Jihyun Keum, Yong Jin Kim, Sae Kyung Choi, Won Moo Lee, Jaeman Bae

**Affiliations:** 1 Department of Obstetrics and Gynecology, College of Medicine, Hanyang University, Seoul 04763, Republic of Korea; goldkjh@hanyang.ac.kr (J.K.); leewmoo@hanyang.ac.kr (W.M.L.); 2 Department of Obstetrics and Gynecology, Korea University Medical College, Seoul 02841, Republic of Korea; zinigo@korea.ac.kr; 3 Department of Obstetrics and Gynecology, College of Medicine, The Catholic University of Korea, Seoul 06591, Republic of Korea; obgysk@catholic.ac.kr

**Keywords:** anti-Müllerian hormone, sex hormone-binding globulin, women with regular menstrual cycles

## Abstract

Background: Polycystic ovarian syndrome (PCOS) can be diagnosed when the anti-Müllerian hormone (AMH) levels are high, but in clinic, women who do not meet the diagnosis of PCOS but have elevated AMH levels are often seen. This study aimed to compare the differences in menstrual cycle patterns and hormone levels in women with regular menstrual cycles, but not PCOS, by dividing them into high and low AMH groups. Material and Methods: This multicenter prospective study included 68 healthy women. Participants with regular menstrual cycles were divided into two groups according to their AMH levels. The main outcome measures were menstrual cycle pattern, body mass index, and hormone levels (thyroid stimulating hormone, prolactin, testosterone, sex hormone-binding globulin, and free androgen index), which were compared between the groups according to AMH levels. The ovulation was assessed by performing pelvic ultrasound, and by assessing the hormone levels of the luteinizing hormone and progesterone. Results: The criteria for determining normal and high AMH levels were based on previous literatures. The participants were divided into normal (39 people) and high (29 people) AMH group. No differences were found in age or BMI between the two groups, and no other differences were observed in TSH, prolactin, testosterone, or free androgen index. However, the high AMH group had significantly higher SHBG levels than the normal group (normal group: 65.46 ± 25.78 nmol/L; high group: 87.08 ± 45.05 nmol/L) (*p* = 0.025). Conclusions: This study is the first to analyze the association between SHBG and AMH levels in women with regular menstrual cycles. Elevated AMH levels are associated with increased levels of SHBG levels.

## 1. Introduction

The anti-Müllerian hormone (AMH), a transforming growth factor-beta (TGF-β), is expressed in the granulosa cells of growing follicles in the ovaries, predominantly in the preantral and small antral follicles [1]. Recently, AMH has been widely utilized for assessing ovarian reserve capacity. AMH is actively used in clinical settings, notably for the treatment of infertility [2]. Additionally, it is useful in the diagnosis of polycystic ovary syndrome (PCOS) [3,4], a prevalent condition affecting approximately 10% of women. It is characterized by various symptoms, including excessive androgen levels, oligomenorrhea or amenorrhea, and metabolic syndrome [5]. Due to its multiple comorbidities, PCOS requires continuous management throughout a woman’s life [1]. The diagnosis of PCOS follows the 2003 Rotterdam criteria [6], but recent studies suggest that an AMH level of 5 or higher is indicative of PCOS [7,8]. In a previous study, more than 97% of women with an AMH level of >10 ng/mL were diagnosed with PCOS; these women demonstrated a higher prevalence of polycystic ovarian morphology and oligomenorrhea compared with those with AMH levels between 5 and 10 ng/mL [4]. However, despite having high AMH levels, some women do not meet the diagnostic criteria for PCOS and experience regular menstrual cycles. Current guidelines discourage the use of AMH alone for the diagnosis of PCOS [9].

This study primarily aimed to identify these characteristics in women with regular menstrual cycles and high AMH levels. In this study, we focused on sex hormone-binding globulin (SHBG) as a diagnostic criterion, along with AMH in PCOS patients [10]. SHBG is a major transport protein of sex hormones, particularly testosterone and estradiol, in the bloodstream. It regulates the concentrations of active sex hormones through high-affinity binding [8]. Previous studies have indicated decreased SHBG levels in metabolic syndrome and PCOS [11].

However, the literature has so far not reported an association between AMH and SHBG in women with regular menstrual cycles, without elevated androgen levels, and without polycystic ovaries.

This study primarily aimed to investigate the length of the menstrual cycle, ovulation rate, and ovulation days in women with high and low AMH levels. The study also aimed to determine the relationship between age, body mass index (BMI), and AMH, which are known to affect AMH, and the association between AMH and SHBG.

## 2. Materials and Methods

### 2.1. Study Population

We enrolled 80 women of reproductive age with regular menstrual cycles. This prospective study was conducted between May 2021 and August 2022 in three tertiary care centers: Hanyang University Hospital (22 participants), Korea University Guro Hospital (27 participants), and the Catholic University of Korea Incheon St. Mary’s Hospital (31 participants). Of the 80 women, only 68 were selected for the study, after excluding 12 women who had clinical hyperandrogenism or polycystic ovarian morphology on ultrasound, or who declined to participate in the clinical study. The participants’ age, height, weight, BMI, menstrual cycle characteristics (cycle length and last menstrual period), and laboratory and ultrasound findings were analyzed.

Women aged 20–45 years with regular menstrual cycles (cycle length: 24–38 days) and who had not used hormone medications (including estrogen-only, progesterone-only, and combined estrogen–progesterone regimens, and levonorgestrel-releasing intrauterine devices) within the past 3 months were included in the study. Conversely, women who met the diagnostic criteria for PCOS (Rotterdam criteria) had elevated total testosterone levels beyond the normal range in blood tests; exhibited clinical manifestations of hyperandrogenism such as acne, hirsutism, or alopecia; were pregnant or breastfeeding; had previously undergone ovarian surgery; or were taking medications for underlying diseases were excluded.

This multicenter study was approved by the respective research ethics committees of the hospitals (IRB Hanyang University Hospital 2020-05-015-007, Korea University IRB no. 2020GR0520, Catholic Incheon ORIB-20200928-025). Informed consent was obtained from all the participants.

### 2.2. Blood Test and Urine Pregnancy Test

The participants underwent a series of five outpatient visits according to their menstrual cycle. The visits were scheduled for the third day of their menstrual cycle, 3 days before ovulation, 1 day before ovulation, 1 day after ovulation, and 3 days after ovulation. A leeway of ±1 day was permitted for each visit. During the initial visit on the third day of the menstrual cycle, assessments included the measurement of AMH, thyroid-stimulating hormone (TSH), prolactin, testosterone, and SHBG levels, and urine pregnancy tests. The participants were officially included in the study after confirming a non-pregnant status. The predicted ovulation day was set to 14 days before the expected start date of the next menstrual period. Blood tests were performed to predict and confirm ovulation. A day prior to ovulation, the luteinizing hormone (LH) and estradiol levels were measured. On the day after ovulation, the LH, estradiol, and progesterone levels were measured to confirm ovulation.

### 2.3. Pelvic Ultrasonography

In each of the five visits, pelvic ultrasound examinations were performed to assess any abnormal findings in the uterus and adnexa, endometrial thickness, the presence of dominant follicles, the size of the dominant follicles, ovulation status, the presence of the corpus luteum, and the presence of fluid in the pelvic cavity. The measurements were performed by a gynecologist at each hospital, with a total of three physicians.

### 2.4. Determination of Ovulation

The presence or absence of ovulation was determined based on the results of pelvic ultrasonography and blood tests performed before and after ovulation. The four pelvic ultrasound findings were combined to determine the presence or absence of ovulation. The determination process was based on a sudden decrease in size or the disappearance of the dominant follicle (at least 18 mm in diameter), the formation of the corpus luteum, the presence of fluid in the pelvic cavity, and a change in the endometrium from a “triple-line appearance” to a “hyperechogenic luteinized” endometrium pattern.

Ovulation, as determined through blood tests, was defined as an increase in the estradiol level (>200 pg/mL) measured 1 day before and 1 day after ovulation [12], a surge in the LH level, and a serum progesterone level of ≥3 ng/mL measured 1 day after ovulation [13]. An ultrasound confirmation of ovulation was performed by two gynecologists.

### 2.5. Determination of Luteal Phase Deficiency

In this study, a luteal phase defect was defined as an inadequate pattern of progesterone secretion from the corpus luteum, associated with infertility and early pregnancy loss. Luteal phase defects were assessed by measuring the length of the luteal phase. This was calculated by determining the actual ovulation date through a pelvic ultrasound and blood test and then subtracting the ovulation date from the menstrual cycle. A luteal phase of less than 11 days was considered indicative of a luteal phase defect [14].

### 2.6. Statistical Analysis

To determine the sample size, we included at least 30 participants in each group to ensure a statistically significant comparison between the experimental and control groups. The results were presented as the mean ± standard deviation (Mean ± SD). Statistical analysis was performed using Statistical Program for Social Sciences (SPSS), version 28.0.1.1. Independent *t*-tests were conducted for normally distributed data, and the non-parametric Mann–Whitney U test was used to analyze non-normally distributed data. Pearson’s correlation analysis was used to perform correlation analysis. A *p*-value of <0.05 was considered significant.

## 3. Results

### 3.1. Demographic Findings

Of the 80 women who participated in this study, 68 were included in the final analysis. Some individuals were excluded for not meeting the criteria, including those with irregular menstrual cycles (outside the range of 25–35 days), who dropped out during the study, with abnormal findings in the initial blood tests, and with PCOS; in total, 12 participants were excluded. The demographic characteristics of the participants are presented in Table 1.

The women’s age distributions were as follows: 20–29 years old: 30.8%; 30–34 years old: 33.8%; 35–40 years old: 27.9%; 41–45 years old: 7.3%. The BMI categories were distributed as follows: underweight (BMI < 18.5): 7%; normal weight (BMI ≥ 18.5 and <23): 58.8%; overweight (BMI ≥ 23 and <25): 16%; obese (BMI ≥ 25): 17%.

Menstrual cycle lengths were categorized as follows: 50% of women had cycles between 25 and 28 days, while 50% had cycles longer than 28 days but shorter than 35 days. The average cycle length was 29.01 days.

The mean values of various blood tests conducted during the initial visit for all women were categorized as follows: AMH: 4.1 ± 2.61 ng/mL; TSH: 1.88 ± 1.02 uIU/mL; prolactin: 13.13 ± 6.33 ng/mL; testosterone: 0.32 ± 0.17 ng/mL; SHBG: 74.68 ± 36.62 nmol/L; free androgen index (FAI): 1.70 ± 1.16.

### 3.2. Comparison of Blood Hormone Levels between Normal and High Groups for Anti-Müllerian Hormone

**Figure 1:**
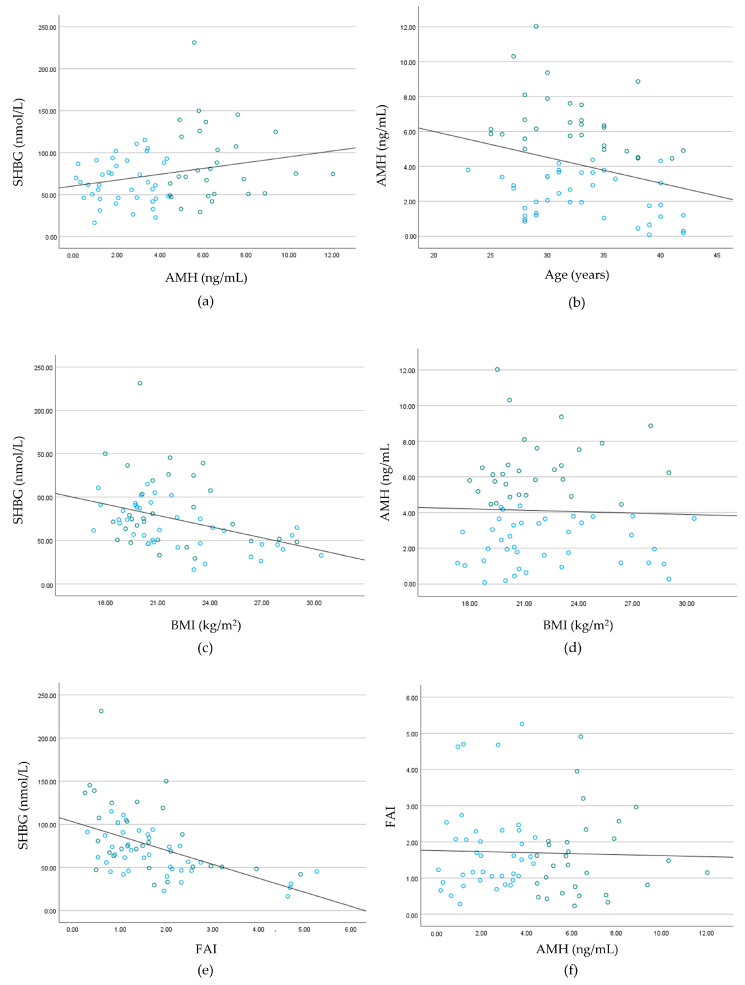
Correlation analysis for each variable. (**a**) Pearson’s correlation analysis of AMH and SHBG (0.247, *p* = 0.042). (**b**) Pearson’s correlation analysis of age and AMH (−0.279, *p* = 0.022). (**c**) Pearson’s correlation analysis of BMI and SHBG (−0.379, *p* = 0.001). (**d**) Pearson’s correlation analysis of BMI and AMH (−0.032, *p* = 0.793). (**e**) Pearson’s correlation analysis of FAI and SHBG (−0.518, *p* = 0.000). (**f**) Pearson’s correlation analysis of AMH and FAI (−0.031, *p* = 0.801). Blue: group with AMH levels < 4.45 ng/mL; green: group with AMH levels > 4.45 ng/mL.

To investigate the association between AMH and SHBG, Pearson’s correlation analysis was performed and revealed a correlation coefficient of 0.247 (*p* = 0.042) (Figure 1a). Given the limited study examining the direct association between AMH and SHBG, a literature review was conducted to establish an appropriate cut-off value for comparing the SHBG levels and clinical manifestations between the normal and high AMH groups. Of these studies, Budiyono et al. [15] analyzed 71 patients with PCOS and 71 normal women aged between 18 and 35 years old and proposed a cut-off value of 4.45 ng/mL for AMH with a sensitivity of 76.1% and a specificity of 74.6% for diagnosing PCOS. Using this method, we classified the participants into a normal (*n* = 39) and a high group (*n* = 29) based on their AMH levels. The characteristics of these groups are presented in Table 2.

The mean BMI values were 22.19 ± 3.5 kg/m^2^ in the normal group, and 21.65 ± 2.85 kg/m^2^ in the high group; no significant difference was found between the groups (*p* = 0.501). The hormonal test results for participants in the normal and high AMH groups were as follows: TSH (normal: 1.81 ± 1.03; high: 1.98 ± 1.01 uIU/mL) (*p* = 0.499), prolactin (normal: 13.31 ± 7.55; high: 12.89 ± 4.28 ng/mL) (*p* = 0.786), total testosterone (normal: 0.311 ± 0.141; high: 0.349 ± 0.205 ng/mL) (*p* = 0.389), and the FAI (normal: 1.79 ± 1.46; high: 1.58 ± 1.26) (*p* = 0.23). No significant differences were observed in these parameters between the groups. However, SHBG was significantly higher in the high group than in the normal group (normal: 65.46 ± 25.78; high: 87.08 ± 45.05 nmol/L) (*p* = 0.025).

### 3.3. Relationship between Menstrual Cycle, Ovulation, and AMH

The length of the menstrual cycle was longer in the high AMH group than in the normal group (normal: 28.5 ± 2.06; high: 29.76 ± 2.44 days) (*p* = 0.025) (Table 3). Ovulation was confirmed based on pelvic ultrasound and blood test findings in all eligible subjects, with no significant differences between the two groups. The lengths from the last menstrual period to ovulation were 15.69 ± 3.20 days in the normal group and 16.33 ± 3.69 days in the high group, showing no significance between the groups (*p* = 0.69).

To determine the presence of luteal phase defects, the length of the luteal phase was measured, which was 12.59 ± 2.71 days in the normal group and 13.3 ± 2.40 days in the high group (*p* = 0.324), with no significance observed between the groups.

### 3.4. Relationship between Age and AMH

The AMH levels decreased as a woman’s age increases (Figure 1b). To eliminate the influence of age factor on the analysis, 35 years was used as the standard age, the point at which ovarian function rapidly decreases [16]. The participants were divided into groups: women aged < 35 years (*n* = 44) and women aged ≥ 35 years (*n* = 24) (Table 4). Comparative analyses were conducted on AMH level, SHGB, menstrual cycle, luteal phase length, and BMI. A significant difference was found in average age between the two groups (group under 35 years old, 29.75 ± 2.72; group over 35 years old, 38.37 ± 2.53 years) (*p* < 0.001). The average AMH level in women aged < 35 years was 4.57 ± 2.65 ng/mL, significantly higher than the figure of 3.24 ± 2.36 ng/mL in women aged ≥ 35 years (*p* = 0.044). No differences were found in BMI and SHBG between the groups according to age (BMI: <35-year age group, 21.76 ± 2.99; ≥35-year age group, 22.34 ± 3.69 kg/m^2^) (n = 0.489) (SHBG: <35-year age group, 77.34 ± 41.64; ≥35-year age group, 69.80 ± 25.02 nmol/L) (n = 0.421).

Women aged < 35 years (*n* = 44) were divided into normal (*n* = 25) and high AMH (*n* = 19) groups. For each group, the AMH, SHBG, menstrual cycle, and luteal phase lengths were compared and analyzed. No difference was found in mean age between groups (normal, 29.92 ± 2.73; high, 29.43 ± 2.92 years) (*p* = 0.595), and the mean AMH levels were 2.64 ± 1.08 (normal) and 7.06 ± 1.95 ng/mL (high), respectively. For SHBG (normal, 61.33 ± 27.26; high, 97.03 ± 50.81 nmol/L) (*p* = 0.006) and menstrual cycle length (normal, 28.62 ± 2.08; high, 30.23 ± 2.80 days) (*p* = 0.045), the high AMH group showed statistically significantly higher levels compared with the normal group. However, no difference was observed in the length of the luteal phase between the two groups (normal, 12.67 ± 3.02; high, 12.96 ± 2.97 days) (*p* = 0.784).

Women aged 35 or older (*n* = 24) were divided into normal (*n* = 14) and high (*n* = 10) AMH groups for comparative analysis (Table 5). No differences were found in age between the two groups (normal, 39.07 ± 2.36; high, 37.40 ± 2.5 years) (*p* = 0.056) for SHBG (normal, 72.83 ± 21.87; high, 65.55 ± 29.57 nmol/L) (*p* = 0.494) and menstrual cycle (normal, 28.28 ± 2.09; high, 28.80 ± 1.68 days) (*p* = 0.528). Additionally, the study by Budi wiweko et al., which is cited in this study to distinguish between the normal and high groups, accounted for the fact that the target group was aged between 18 and 35 years old, and the same level of AMH was found in the high group of women aged ≥ 35 years. However, their applicability is limited. Therefore, the AMH level had a cut-off value of 3.72 ng/mL (95% confidence interval: 3.55–3.80; positive predictive value: 0.86; negative predictive value: 0.96) and was re-analyzed [17]. In these figures, women aged ≥ 35 years (*n* = 24) were divided into normal (*n* = 12) and high AMH groups (*n* = 12) for comparative analysis (Table 5). The AMH levels (normal, 1.24 ± 1.06; high, 5.24 ± 1.36 ng/mL) (*p* = 0.00) and age (normal, 39.33 ± 2.22; high, 37.42 ± 2.54 years) (*p* = 0.062) were assessed. No significant difference was found in the SHBG (normal, 72.13 ± 67.47; high, 67.47 ± 27.95 nmol/L) (*p* = 0.659). However, the menstrual cycle length was significantly longer in the high AMH group (normal, 27.67 ± 1.23; high, 29.33 ± 2.14 days) (*p* = 0.032). The results of SHBG showed no difference depending on the level of AMH at both cut-off values in the group aged ≥ 35 years.

### 3.5. Body Mass Index and Anti-Müllerian Hormone Levels

According to the World Health Organization guidelines, the normal weight standard for Asian people is 23. The participants were segregated into normal (*n* = 45) and high BMI (*n* = 23) groups (Table 6). The average BMI value was 19.98 ± 1.23 kg/m^2^ in the normal BMI group and 25.76 ± 2.38 kg/m^2^ in the high BMI group. No difference was found in average age between the two groups (normal BMI group, 28.75 ± 2.1 years; high BMI group, 29.54 ± 2.66 years) (*p* = 0.191). However, the AMH level was significantly higher in the high BMI group (normal BMI group, 2.81 ± 1.98; high BMI group, 6.53 ± 1.90 ng/mL) (*p* < 0.001). The SHBG levels (normal BMI group, 73.19 ± 37.5; high BMI group, 74.74 ± 33.80 nmol/L) (*p* = 0.869) and menstrual cycle length (normal BMI group, 28.75 ± 2.1; high BMI group, 29.54 ± 2.66 days) (*p* = 0.191) showed no significant differences according to BMI categories. To eliminate the influence of BMI-related factors, the normal (*n* = 45) and high (*n* = 23) BMI groups were further stratified into normal and high AMH groups, respectively. In the normal BMI group (*n* = 45), the normal AMH (*n* = 39) and high AMH (*n* = 5) subgroups were analyzed. Notably, the high AMH subgroup demonstrated significantly higher values for BMI (normal group, 19.19 ± 0.83; high group, 22.08 ± 0.37 kg/m^2^) (*p* < 0.001), SHBG (normal group, 62.48 ± 27.35; high group, 133.46 ± 60.67 nmol/L) (*p* < 0.001), and menstrual cycle length (normal group, 28.55 ± 2.07; high group, 30.70 ± 1.30 days) (*p* = 0.035). Subsequently, within the high BMI group consisting of 23 individuals, the normal AMH (*n* = 15) and high AMH groups (*n* = 8) were analyzed. Notably, the BMI (normal group, 26.22 ± 2.43; high group) showed no difference in age (normal group, 33.07 ± 5.86; high group, 33.77 ± 5.69 years) (*p* = 0.8) between the two groups. However, the high AMH subgroup exhibited significantly higher SHBG levels (normal group, 44.83 ± 17.70; high group, 78.53 ± 38.33 ng/mL) (*p* = 0.009) and menstrual cycle length (normal group, 28.85 ± 2.38; high group, 30.70 ± 1.38 days) (*p* = 0.041). Considering the acknowledged impact of BMI on SHBG levels, the results of our analysis of all participants revealed a negative correlation between BMI and SHBG (Pearson’s correlation analysis −0.379, *p* = 0.001) (Figure 1c). Pearson’s correlation analysis, accounting for the effects of age and BMI, demonstrated a correlation of 0.246 (*p* = 0.046) between AMH and SHBG.

### 3.6. Endometrial Thickness, Dominant Follicle Size, and Anti-Müllerian Hormone Levels

The thickness values of the endometrium measured via pelvic ultrasound were 12.49 ± 2.79 mm and 12.12 ± 2.02 mm in the normal and high AMH groups, respectively, with no significant differences recorded between the two groups (*p* = 0.567) (Table 7). Given the importance of the presence or absence of an appropriately sized dominant follicle for ovulation, we compared the sizes of the largest follicles of the dominant follicles measured before and after ovulation. The follicle’s size was 19.85 ± 4.64 mm in the normal AMH group and 18.01 ± 4.10 mm in the high AMH group. No significant difference was observed between the two groups (*p* = 0.112).

## 4. Discussion

This study aimed to determine the association between AMH levels, SHBG levels, ovulation rate, and luteal phase length in women with regular menstrual cycles. The cut-off values suggestive of PCOS were used based on previous literature studies; in this study, we categorized women with relatively high and low AMH levels and compared the clinical features and hormonal levels of these groups. Among women < 35 years, those with relatively high AMH levels showed increased SHBG levels compared with those with low AMH levels.

Currently, AMH is actively used in clinical practice [18]. AMH is a dimeric glycoprotein of the transforming growth factor- β (TGF-β) superfamily and expressed in granulosa cells of growing follicles in the ovary, predominantly in preantral and small antral follicles [1]. After the final stage of follicular growth, AMH is no longer expressed under the influence of follicle-stimulating hormone (FSH). Because of these characteristics, AMH is actively used in clinical practice to evaluate ovarian reserve and response to infertility treatment. In addition, PCOS is characterized by an increased number of follicles, and the existing literature strongly supports the use of AMH levels in the diagnosis of PCOS [10,15,19,20]. PCOS is a prevalent female disorder affecting approximately 10% of women, although its exact pathophysiology remains unknown. The clinical manifestations include anovulation, hyperandrogenism, and polycystic ovaries as observed on ultrasound [20]. The diagnosis of PCOS is established based on the Rotterdam diagnostic criteria (2003) and is possible when two or more of the abovementioned clinical features are satisfied [21]. Numerous studies propose the use of AMH levels for diagnosing POCS. Fallat et al. [22] noted a twofold to threefold increase in AMH levels in women with PCOS compared with those with normal ovaries, while other data suggested a threshold of 5 or higher [23]. Various studies have suggested different cut-off values; Budi et al. reported a threshold of 4.45 ng/mL [15], while Lin et al. stated that PCOS can be diagnosed when the level is 4 ng/mL or higher [24]. Yue et al. [25] reported AMH levels of 8.16 ng/mL between 20 and 29 years of age and 5.89 ng/mL between 30 and 39 years of age.

However, AMH is influenced by various factors, such as age [7] and BMI [26], making its consistent application in clinical practice challenging. Additionally, despite not meeting the diagnostic criteria for PCOS, many women in our clinic exhibited AMH levels exceeding those indicated in the existing literature. Therefore, this study aimed to compare the clinical features and hormonal levels of women with regular menstrual cycles and those without typical clinical features of PCOS based on varying AMH levels.

Our study attempted to include women of childbearing age with regular menstruation. To exclude potential causes of menstrual cycle irregularities, the TSH, prolactin, and total testosterone were measured. The values of all participants were normal. The average values were also within the normal range (Table 1). When classified according to AMH levels, no differences were observed in the average TSH, prolactin, and testosterone levels between the groups (Table 2). The primary hallmark of PCOS is hyperandrogenemia, which is diagnosed through clinical and biochemical assessments [27]. The biochemical markers utilized for diagnosing hyperandrogenism include total and free testosterone, androstenedione, dehydroepiandrosterone sulfate, and FAI [28,29]. In this study, the FAI was also examined to confirm the presence of hyperandrogenism, revealing no significant difference between the AMH groups (Table 2, Figure 1f).

SHBG is a plasma glycoprotein synthesized in the liver that functions as a carrier of sex hormones. SHBG binds to circulating sex hormones, thereby elevating the concentrations of freely available sex hormones in their active forms within the bloodstream. Its pivotal role in the regulatory processes is illustrated in Figure 1e [30]. A reduction in SHGB has previously been correlated with certain conditions, such as metabolic syndrome, type 2 diabetes [31], cardiovascular disease [32], and PCOS [23]. Consequently, Calzada et al. proposed combining SHBG and AMH for diagnosing PCOS, suggesting threshold values of 5.17 ng/mL for AMH and 38.5 nmol/L for SHBG [10].

Based on these previous studies, our study hypothesized that SHBG might play a crucial role in women exhibiting elevated AMH levels, surpassing the standard considered indicative of PCOS, yet devoid of clinical PCOS signs. In this study, we compared SHBG concentrations in women with regular menstrual cycles, stratifying them into high and low AMH groups (Figure 1a). The SHBG levels were 65.46 ± 25.78 (normal group) and 87.08 ± 45.05 nmol/L (high group) (Table 2), being significantly higher in women with high AMH levels (Figure 1a). This pattern of SHBG increase with AMH level elevation persisted even when accounting for age and BMI, both known factors affecting SHBG (Table 4 and Table 6). However, in women aged > 35 years, no significant difference was found in SHBG levels according to the AMH levels (Table 5). Based on the literature review, the direct correlation between AMH and SHBG remains unclear. Considering the substances influencing the synthesis of each protein, AMH is secreted from the granulosa cells of the ovary, and its expression is regulated by estradiol, LH, FSH, and androgens [33]. SHBG expression occurs through the peroxisome proliferator-activated receptor γ (PPAR γ) via the estrogen receptor-alpha (ER-α) and estrogen receptor-beta (ER-β) of estradiol. This expression is activated by inhibition [34]. Consequently, both AMH and SHBG protein syntheses are affected by estradiol, but their direct mechanisms have not yet been elucidated. Therefore, further studies into the tendency for higher levels of SHBG in women with high AMH levels and with regular menstrual cycles are needed. Furthermore, when exclusively focusing on women aged > 35 years, no significant difference was observed in SHBG between the groups (Table 5). Given the limited sample size of 68 women, with 24 aged > 35 years, the statistical analysis was constrained; thus, the results must be validated through future large-scale studies. Moreover, FAI, which displays a distinct negative correlation with SHBG, might have clinical significance for PCOS (Figure 1e). Therefore, it is essential to investigate the relationship between FAI and AMH. Pearson’s correlation analysis was conducted, but no significant correlation was identified (Pearson’s correlation analysis: −0.031, *p* = 0.801) (Figure 1f).

Another noteworthy point is highlighted in Table 6, where the average AMH level for individuals with a BMI of 23 or more is 6.53 ± 1.90 ng/mL, surpassing the AMH level for women with normal weight (2.81 ± 1.98 ng/mL). This finding contrasts with those of previous studies on BMI and AMH, where the hormone level tended to decrease when the BMI was 30 or more [29]. Notably, among the participants in this study, only one woman had a BMI of ≥30. The participants were categorized according to the World Health Organization standard for Asian obesity, which is 25. We inferred that this participant distribution contributed to the absence of an AMH decline corresponding to an increase in BMI (Pearson’s correlation analysis: −0.032, *p* = 0.793) (Figure 1d). In this study, the SHBG levels tended to decrease as BMI increased (Pearson’s correlation: −0.379, *p* = 0.001) (Figure 1c). However, when comparing averages by dividing the participants into normal and high BMI groups, no significant differences were observed in SHBG levels (Table 6). This finding suggests that factors other than BMI may influence SHBG; therefore, it is crucial to consider the large standard deviation of SHBG when interpreting these results.

The primary clinical presentation of PCOS is anovulation-associated amenorrhea or oligomenorrhea. In this study, we hypothesized that the group with elevated AMH levels would exhibit anovulation, a characteristic of PCOS. However, all participants exhibited ovulation (Table 4). The inclusion of women with regular menstrual cycles in this study underscores the caution needed in presuming PCOS solely based on elevated AMH levels. Nevertheless, the observation that the group with high AMH levels had a longer menstrual cycle than the group with normal AMH aligns with the results of previous studies [29].

In addition, observing the presence or absence of dominant follicles using pelvic ultrasonography is a clinically valuable method for confirming ovulation. The likelihood of ovulation increases in dominant follicles at least 18 mm in length. The maximum diameter of the dominant follicle did not differ significantly between the two groups (Table 7). Furthermore, PCOS is clinically significant because of the potentially increased risk of miscarriage during early pregnancy associated with luteal phase defects [35]. However, endometrial biopsy, which measures luteal phase defects, is rarely performed in clinical settings. In this study, the assessment of luteal phase length, following the method outlined by Ginsburg et al. [36], revealed no significant difference between the two groups. This observation suggests that normal luteal phases were prevalent (Table 3). Such findings emphasize the caution needed in diagnosing PCOS based solely on the AMH levels in women with regular menstrual cycles.

Further studies are necessary to determine the cause of the increased SHBG levels in women with high AMH levels during a regular menstrual cycle. In PCOS, although AMH levels are elevated, menstrual cycle abnormalities such as amenorrhea or oligomenorrhea are common, and SHBG tends to decrease [10]. The expression of SHBG is influenced by several factors. Individual gene expression is a crucial endogenous factor, and genes are located on the short arm (p-arm) of chromosome 17. Genetic polymorphisms such as Rs6259, Rs6258, and Rs727428(TAAAA)(n) have been previously identified [33]. Several factors affect the expression of SHBG. When monosaccharides such as glucose or fructose increase, the level of hepatocyte nuclear factore-4α, which plays an important role in regulating the SHBG promotor, is reduced, thereby inducing lipogenesis. This consequently reduces the expression of SHBG [37]. Drugs can also affect SHBG protein expression, with representative known medications including metformin [35], oral contraceptives [38,39], and myoinositol [40]. In particular, these drugs were identified through research on PCOS patients, where they are commonly employed as part of PCOS treatment [38]. Recognizing the potential impact of these medications on the study results, our investigation focused on women who stopped taking the medication for >3 months.

**Figure 2:**
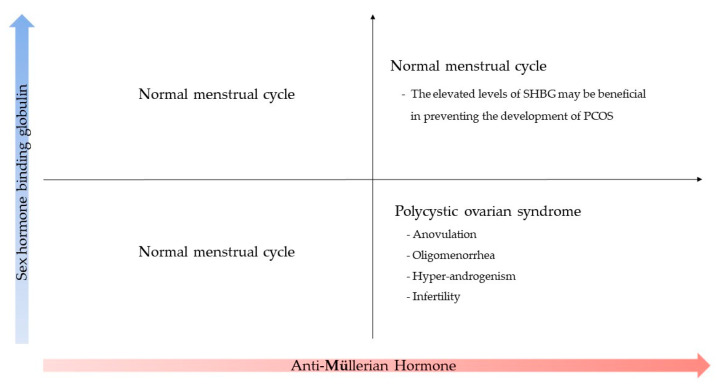
Relationship between the concentrations of AMH and SHBG and women’s menstrual cycles. SHBG may prevent the development of PCOS in women with high AMH levels.

Considering the results of this study, some unexplained factors contribute to the elevation of SBHG levels in women with high AMH levels. This tendency is believed to prevent the development of PCOS (Figure 2).

## 5. Conclusions

In conclusion, among women with regular menstrual cycles, those with elevated AMH levels exhibited higher SHBG levels than those with relatively low AMH levels. Additionally, women with high AMH levels generally have longer menstrual cycles, normal ovulation, and the absence of luteal phase defects. Future studies should focus on molecular biological investigations to identify SHBG levels based on AMH concentrations. Additionally, large-scale comparative studies of patients diagnosed with PCOS are warranted.

## Figures and Tables

**Table 1:** Characteristics of the study participants.

Variables	Number (*n* = 68)	Percentage	Mean	SD
Age (years)				
20–29	21	30.8		
30–34	23	33.8		
35–40	19	27.9		
41–45	5	7.3		
BMI (kg/m^2^)			21.97	3.24
<18.5	5	7		
18.5–22.9	40	58.8		
23–25	11	16		
>25	12	17		
Menstrual cycle length (days)			29.01	2.29
≤28	34	50		
>28	34	50		
AMH (ng/mL)			4.1	2.61
TSH (uIU/mL)			1.88	1.02
Prolactin (ng/mL)			13.13	6.33
Testosterone (ng/mL)			0.32	0.17
SHBG (nmol/L)			74.68	36.62
FAI			1.7	1.16

**Table 2:** Comparison of demographic and hormone levels between normal AMH and high AMH groups.

Variables	Number AMH (<4.45) (*n* = 39)	High AMH (≥45) (*n* = 39)	*p* Value
AMH (ng/mL)	2.28 ± 1.28	6.55 ± 1.81	<0.001
Age (years)	33.2 ± 5.14	32.24 ± 4.63	0.428
BMI (kg/m^2^)	22.19 ± 3.51	21.65 ± 2.85	0.501
TSH (uIU/mL)	1.81 ± 1.03	1.98 ± 1.01	0.499
Prolactin (ng/mL)	13.31 ± 7.55	12.89 ± 4.28	0.786
Testosterone (ng/mL)	0.311 ± 0.141	0.349 ± 0.205	0.389
SHBG (nmol/L)	65.46 ± 25.78	87.08 ± 45.05	0.025
FAI	1.79 ± 1.46	1.58 ± 1.26	0.23

**Table 3:** Evaluation of the menstrual cycle, ovulation rate, and luteal phase length between normal AMH and high AMH groups.

Variables	Normal AMH (*n* = 39)	High AMH (*n* = 29)	*p* Value
Menstrual cycle (days)	28.5 ± 2.06	29.76 ± 2.44	0.025
Ovulation rate (%)	1 ± 0.00	1 ± 0.00	
Expected ovulation day (MCD)	15.69 ± 3.20	16.33 ± 3.69	0.69
The length of luteal phase (days)	12.59 ± 2.71	13.3 ± 2.40	0.324

**Table 4:** Comparison of variables considering age.

**Variables**	**Age (<35) (*n* = 44)**	**Age (≥35) (*n* = 24)**	***p*** **-Value**
Age (years)	29.75 ± 2.72	38.37 ± 2.53	<0.001
BMI (kg/m^2^)	21.76 ± 2.99	22.34 ± 3.69	0.489
AMH (ng/mL)	4.57 ± 2.65	3.24 ± 2.36	0.044
SHBG (nmol/L)	77.34 ± 41.64	69.80 ± 25.02	0.421
**Below 35 years old (*n* = 44)**			
**Variables**	**Normal AMH (*n* = 25)**	**High AMH (*n* = 19)**	***p*** **-Value**
Age (years)	29.92 ± 2.73	29.43 ± 2.92	0.595
AMH (ng/mL)	2.64 ± 1.08	7.06 ± 1.95	<0.001
SHBG (nmol/L)	61.33 ± 27.26	97.03 ± 50.81	0.006
Menstrual cycle (days)	28.62 ± 2.08	30.23 ± 2.80	0.045
Length of luteal phase (days)	12.67 ± 3.02	12.96 ± 2.97	0.784

**Table 5:** Comparison of variables considering age (≥35 years old) (*n* = 24).

**Variables**	**Normal AMH (<4.45) (*n* = 14)**	**High AMH** **(≥4.45) (*n* = 10)**	***p*** **-Value**
Age (years)	39.07 ± 2.36	37.40 ± 2.54	0.056
AMH (ng/mL)	1.64 ± 1.41	5.48 ± 1.36	<0.001
SHBG (nmol/L)	72.83 ± 21.87	65.55 ± 29.57	0.494
Menstrual cycle (days)	28.28 ± 2.09	28.80 ± 1.68	0.528
Length of luteal phase (days)	12.46 ± 2.18	13.87 ± 0.86	0.51
**Variables**	**Normal AMH (<3.72) (*n* = 12)**	**High AMH** **(≥3.72) (*n* = 12)**	***p*-Value**
Age (years)	39.33 ± 2.22	37.42 ± 2.54	0.062
AMH (ng/mL)	1.24 ± 1.06	5.24 ± 1.36	0
SHBG (nmol/L)	72.13 ± 67.47	67.47 ± 27.95	0.659
Menstrual cycle (days)	27.67 ± 1.23	29.33 ± 2.14	0.032

**Table 6:** Comparison of variables by BMI groups.

**Variables**	**Normal BMI (<23) (*n* = 45)**	**High BMI (≥23) (*n* = 23)**	***p*-Value**
BMI (kg/m^2^)	19.98 ± 1.23	25.76 ± 2.38	<0.001
Age (years)	28.75 ± 2.10	29.54 ± 2.66	0.191
AMH (ng/mL)	2.81 ± 1.98	6.53 ± 1.90	<0.001
SHBG (nmol/L)	73.19 ± 37.50	74.74 ± 33.80	0.869
Menstrual cycle (days)	28.75 ± 2.10	29.54 ± 2.66	0.191
**BMI < 23 (*n* = 45)**			
**Variables**	**Normal AMH (<4.45) (*n* = 39)**	**High AMH (≥4.45) (*n* = 6)**	***p*-Value**
BMI (kg/m^2^)	19.19 ± 0.83	22.08 ± 0.37	<0.001
AMH (ng/mL)	2.58 ± 1.10	6.95 ± 1.52	<0.001
Age (years)	30.11 ± 2.86	29.00 ± 2.91	0.432
SHBG (nmol/L)	62.48 ± 27.35	133.46 ± 60.67	<0.001
Menstrual cycle (days)	28.55 ± 2.07	30.70 ± 1.30	0.035
**BMI (≥23) (*n* = 23)**			
**Variables**	**Normal AMH (<4.45) (*n* = 15)**	**High AMH (≥4.45) (*n* = 8)**	***p*-Value**
BMI (kg/m^2^)	26.22 ± 2.43	25.05 ± 2.26	0.264
AMH (ng/mL)	2.32 ± 1.25	6.86 ± 1.68	<0.001
Age (years)	33.07 ± 5.86	33.77 ± 5.69	0.8
SHBG (nmol/L)	44.83 ± 17.70	78.53 ± 38.33	0.009
Menstrual cycle (days)	28.85 ± 2.38	30.70 ± 1.38	0.041

**Table 7:** Sonographic findings in women with normal AMH levels and those with high AMH levels.

Variables	Normal AMH (*n* = 36)	High AMH (*n* = 26)	*p*-Value
Endometrial thickness (mm)	12.49 ± 2.79	12.12 ± 2.02	0.567
Largest dominant follicle diameter (mm)	19.85 ± 4.64	18.01 ± 4.10	0.112

## Data Availability

The data presented in this study are available on request from the corresponding author.
